# HIV testing, HIV status and outcomes of treatment for tuberculosis in a major diagnosis and treatment centre in Yaounde, Cameroon: a retrospective cohort study

**DOI:** 10.1186/1471-2334-12-190

**Published:** 2012-08-15

**Authors:** Eric Walter Pefura Yone, Christopher Kuaban, André Pascal Kengne

**Affiliations:** 1 Department of Internal Medicine and Subspecialties, Faculty of Medicine and Biomedical Sciences, University of Yaounde I, Yaounde, Cameroon; 2 Pneumology service, Yaounde Jamot Hospital, Yaounde, Cameroon; 3 South African Medical Research Council & University of Cape Town, Cape Town, South Africa; 4 Yaounde Jamot’s Hospital, P.O Box: 4021, Yaounde, Cameroun

**Keywords:** Tuberculosis, HIV infection, Outcomes

## Abstract

**Background:**

Human immuno-deficiency virus (HIV) infection and tuberculosis are common and often co-occurring conditions in sub-Saharan Africa (SSA). We investigated the effects of HIV testing and HIV status on the outcomes of tuberculosis treatment in a major diagnosis and treatment centre in Yaounde, Cameroon.

**Methods:**

Participants were 1647 adults with tuberculosis registered at the Yaounde Jamot’s Hospital between January and December 2009. Multinomial logistic regression models were used to relate HIV testing and HIV status to the outcomes of tuberculosis treatment during follow-up, with adjustment for potential covariates.

**Results:**

Mean age of participants was 35.5 years (standard deviation: 13.2) and 938 (57%) were men. Clinical forms of tuberculosis were: smear-positive (73.8%), smear-negative (9.4%) and extra-pulmonary (16.8%). Outcomes of tuberculosis treatment were: cure/completion (68.1%), failure (0.4%), default (20.1%), death (5.2%) and transfer (6.3%). Using cure/completion as reference, not testing for HIV was associated with adjusted odds ratio of 2.30 (95% confidence interval: 1.65-3.21), 2.26 (1.29-3.97) and 2.69 (1.62-4.46) for the risk of failure/default, death and transfer respectively. The equivalents for a positive test among those tested (1419 participants) were 1.19 (0.88-1.59), 6.35 (3.53-11.45) and 1.14 (0.69-1.86).

**Conclusions:**

Non-consent for HIV testing in this setting is associated with all unfavourable outcomes of tuberculosis treatment. However been tested positive was the strongest predictor of fatal outcome. Efforts are needed both to improve acceptance of HIV testing among patients with tuberculosis and optimise the care of those tested positive.

## Background

Human immunodeficiency virus (HIV) infection and tuberculosis are major health problems in sub-Saharan Africa (SSA). In 2009 alone, 35% of the global 9.4 millions declared cases of tuberculosis (TB) were registered in Africa. In that same year, the rate of TB/HIV co-infection at the global level was 11–13%, but was as high as 80% in Africa [1]. HIV infection favours the reactivation and progression of latent *Mycobacterium tuberculosis* infection to overt tuberculosis infection, and *Mycobacterium tuberculosis* favours the replication of HIV and precipitates the natural course of the infection toward advanced stage or severe immune-depression [2,3]. Once tuberculosis becomes clinically manifest, co-infection with HIV is generally associated with poor outcome in SSA [4]. TB/HIV co-infection has been well-documented in SSA with regard to smear-positive pulmonary tuberculosis [5-8]. The changing pattern with time has been described in places as well. In Cameroon for instance, the prevalence of HIV infection among those with smear-positive pulmonary tuberculosis increased from 16.6% in 1997 to about 29.3% in 2007 [5,9]. However, little is known about the prevalence of HIV in other clinical form of tuberculosis, as well as effects of HIV on the broad categories of outcomes of care for tuberculosis.

The current study has assessed the prevalence of HIV infection among African adults with tuberculosis, regardless of the clinical form, and quantified the effects of failure to test for HIV, and HIV status on the outcome of tuberculosis treatment in a major diagnosis and treatment centre in Cameroon.

## Methods

### Study setting

The study was conducted in the pneumology service of Yaounde Jamot’s Hospital (YJH). The YJH serves a referral centre for tuberculosis and respiratory diseases for the capital city of Cameroon (Yaounde) and surrounding areas as described in detail elsewhere [10]. It is one of the major centres for diagnosis and treatment of tuberculosis (CDT) in Cameroon, and YJH also host an approved treatment centre for people living with HIV infection. From year 2006 to 2011, about 1600 to 1800 patients with tuberculosis were diagnosed and treated on an annual basis in this CDT. In the year 2009, up to 11% of all cases of tuberculosis diagnosed in the country were managed at the YJH. Patients received at the CDT during January to December 2009 were considered for inclusion in the study. The study was approved by the institutional review board of the YJH.

### Definition and classification of tuberculosis cases

Patients who receive care at the CDT of YJH are consecutively registered as they are started on treatment. For those with a past exposure to antituberculosis treatment, the approach is nearly similar. Patients who report back to the centre with active tuberculosis and who have been treated in the past for at least one month are registered again with a new number and started on a standardised re-treatment regimen. The following international definitions are applied: [2,3,9] 1) smear-positive pulmonary tuberculosis (PTB+) - acid-fast bacilli (AFB) found in at least two sputum specimens; 2) smear-negative pulmonary tuberculosis (PTB-) – persisting negativity on three sputum examinations after ten-day course of non-specific antibiotic treatment in a patient with tuberculosis-like clinical and radiological signs, and in the absence of any obvious cause; 3) extra-pulmonary tuberculosis (EPTB) – tuberculosis involving organs other than the lungs. Patients with past exposure to antituberculosis treatment are usually all smear-positive and are further classified as “relapse” (i.e. reoccurrence of the disease following a successful antituberculosis treatment course), “failure” (i.e. positive smear after five months of antituberculosis treatment) and “treatment after default” (i.e. starting antituberculosis treatment again after two consecutive months of interruption). A “new case” is a patient with tuberculosis who has never been exposed to antituberculosis treatment for more than one month in the past. “Other cases of tuberculosis” referred to patients who cannot fit in one of the categories described above.

### Detection and management of HIV infection

At the CDT of the YJH, all patients with tuberculosis are screened for HIV infection free of charge after informed consent has been obtained from the patient or relative for dependant patients. This includes detection of anti-HIV 1 and anti-HIV 2 antibodies in the serum with the use of two rapid tests: Determine® HIV ½(Abbot laboratories, Tokyo, Japan) and Immunocomb® II HIV 1 and 2 Bispot (Organics, Courbevoie, France). A patient is classified as HIV positive when the two tests are positive. For discordant tests, a confirmatory western blot test (New Lav Blot, Sanofi diagnostics-Pasteur) is conducted. All HIV-positive patients are started on prophylaxis with cotrimoxazole and those with CD4 count <200/mm^3^ are started on highly active antiretroviral therapy free of charge. Initial antiretroviral regimens are the combinations lamivudine-zidovudine-efavirenz or lamivudine-stavudine-efavirenz.

### Tuberculosis treatment

Tuberculosis treatment at the CDT is made according to the guidelines of the Cameroon National Programme Against Tuberculosis (NPAT) and the WHO recommendations [11,12]. Patients are either admitted during the intensive phase of antituberculosis treatment, or treated as outpatient. Antituberculosis drugs are dispensed free of charge to all patients. Treatment regimens used are standard regimens of category I for new patients and of category II for re-treatment cases. New cases are treated with a regimen that includes an intensive phase of two months duration with rifampicin (R), isoniazid (H), ethambutol (E) and pyrazinamide (Z), followed by a 4-month continuation phase with rifampicin and isoniazid (2RHEZ/4RH). During re-treatment, category I medications (R, H, E, Z) are completed with streptomycin (S). Therefore, re-treatment cases are treated with RHEZS for two months, followed by one month on RHEZ and five months on RHE (2RHEZS/1RHEZ/5RHE). During the intensive phase, adherence is directly monitored by the healthcare team for patients admitted, and during weekly drug collection in those treated as outpatients. The continuation phase is conducted on the outpatient basis and adherence assessed during monthly drugs collection visits.

### Monitoring and outcomes of tuberculosis treatment

During antituberculosis treatment, PTB+ patients are re-examined for AFB at the end of month 2, 5 and 6 for new cases, and at the end of month 3, 5 and 8 in case of re-treatment. PTB- and patients with EPTB are monitored clinically and/or radiologically at the same frequency. At the end of the treatment, patients are ranked into mutually exclusive categories [13] as: 1) cured – patient with negative smear at the last month of treatment and at least one of the preceding; 2) treatment completed – patient who has completed the treatment and for whom the smear result at the end of the last month is not available; 3) failure – patient with positive smear at the 5^th^ month or later during treatment; 4) death – death from any cause during treatment; 5) defaulter – patient who’s treatment has been interrupted for at least two consecutive months; 6) transfer – patient transferred to complete his treatment in another centre and who’s treatment outcome is unknown.

### Data collection

This was a retrospective cohort study among TB patients aged ≥ 15 years. Tuberculosis treatment registers and antituberculosis treatment forms of the YJH’s CDT served as basis for data collection for the study. Data were collected on age, sex, residence (urban vs. rural), history of exposure to antituberculosis treatment, localisation of tuberculosis infection, status for HIV infection, CD4 lymphocyte count (in those with HIV infection) and outcome of antituberculosis treatment. Cured and antituberculosis treatment completion were considered as favourable outcome (successful treatment) while death, default and failure were considered as unfavourable outcome [14].

### Statistical methods

Data analysis used SPSS® v.12.0.1 for Windows® (SPSS Inc., Chicago, USA) and SAS/STAT® v 9.1 for windows (SAS Institute Inc., Cary, NC, USA). Results are presented as count (proportions), means (standard deviation, SD) or median (interquartile range, IQR). Group comparisons used chi square of Fisher exact test for qualitative variables, and Student t-test or Mann-Whitney U test for quantitative variables. Multinomial Logistic regression models were used to investigate the effects of HIV testing and HIV status on the outcomes of tuberculosis in sex and age adjusted analysis, and after further adjustment for other potential confounders. A p-value < 0.05 was used to characterise statistically significant results.

## Results

### Study population

During the year 2009, 1647 patients were screened and treated for tuberculosis at the Yaounde Jamot Hospital’s Centre for Diagnosis and Treatment. They were aged 33 years (interquartile range: 25-43 years) and 938 (57%) were men. Clinical forms of tuberculosis were: 1216 (73.8%) for PTB+, 155 (9.4%) for PTB- and 276 (16.8%) for PTB (Table 1).

**Table 1:** Characteristics of patients with tuberculosis according to the treatment outcome at the Jamot Hospital in 2009

**Characteristics**	**Categories**	**Total**	**Outcomes of tuberculosis treatment**					
			Cured/Completed*	**Failure**	**Deaths**	**Defaulted**	**Transferred**	**P-value**
**N(%)**		1647	1121 (68.1)	6 (0.4)	86 (5.2)	331 (20.1)	103 (6.3)	
**Age, years, n(%)**								
	≤33	854 (51.9)	609 (71.3)	2 (0.2)	24 (2.8)	172 (20.1)	47 (5.5)	<0.001
	>33	793 (48.1)	512 (64.6)	4 (0.5)	62 (7.8)	159 (20.1)	56 (7.1)	
	Mean (SD)	35.5 (13.2)	35.3 (12.9)	37.3 (13.7)	43.9 (14)	35.7 (13.3)	37.1 (13.9)	<0.001
**Men, n (%)**		938 (57)	635 (67.7)	4 (0.4)	50 (5.3)	194 (20.7)	55 (5.9)	0.467
**Urban residence, n (%)**		1390/1622 (85.7)	968 (69.6)	5 (0.4)	72 (5.2)	278 (20)	67 (4.8)	<0.001
**Clinical forms, n (%)**								
	PTB+	1216 (73.8)	845 (69.5)	6 (0.5)	56 (4.6)	239 (19.7)	70 (5.7)	<0.001
	PTB-	155 (9.4)	86 (55.5)	0 (0)	16 (10.3)	42 (27.1)	11 (7.1)	
	EPTB	276 (16.8)	190 (68.8)	0 (0)	14 (5.1)	50 (3.7)	22 (8.0)	
**Type of patient, n (%)**								
	New cases	1505 (91.4)	1026 (68.2)	5 (0.3)	81(5.4)	295 (19.6)	98 (6.5)	0.27
	Relapse	119 (7.2)	81(68.1)	1 (0.8)	5 (4.2)	28 (23.5)	4 (3.4)	
	Failure	3 (0.2)	2 (66.7)	0 (0)	0 (0)	0 (0)	1 (33.3)	
	Defaulters	20 (1.2)	12 (60)	0 (0)	0 (0)	8 (40)	0 (0)	
**HIV serology, n (%)**								
	Not done	228 (13.8)	113 (49.6)	2 (0.9)	20 (8.8)	69 (30.3)	24 (10.5)	<0.001
	Negative	922/1419 (65)	685 (74.3)	4 (0.4)	17 (1.8)	166 (18)	50 (5.4)	<0.001
	Positive	497/1419 (35)	323 (65.0)	0 (0)	49 (9.8)	96 (19.3)	29 (5.8)	

SD, standard deviation; EPTB, extra-pulmonary tuberculosis ; PTB+, smear positive pulmonary tuberculosis ; PTB-, smear negative pulmonary tuberculosis; cured + completed = therapeutic success.

### Outcomes of TB

In all, 272 (16.5%) patients were cured and 849 (51.5%) successfully completed the treatment, giving and overall treatment success rate of 68.1%. Other outcomes of care were failure (0.4%), death (5.2%), default (20.1%) and transfer (6.1%). Characteristics of participants according to the outcomes of care are described in Table 1, showing some significant differences in the distribution by residence (urban vs. rural) and clinical forms of tuberculosis.

### HIV testing and status

A total 1419 (86.2%) patients were tested for HIV, and 497 (35%) were positive for HIV, all for HIV-1 subtype. Of those tested positive, 229 (46.1%) received antiretroviral therapy. Compared with those who did not test, patients who tested for HIV were borderline younger (35.3 vs. 37.0 years, p = 0.06), included more women (44.6% vs. 33.3%, p = 0.001), and more individuals with smear positive tuberculosis than with smear negative or extra-pulmonary tuberculosis (75.8%, 8%, 16.2% vs. 61.8%, 18% and 20.2%, p < 0.001). Among participants tested for HIV, patients with positive status as compared with the negative ones were older (36 vs. 30 years, p < 0.001), included more women (57.1% vs. 37.3%, p < 0.001), included fewer patients with smear positive than with smear negative and extra-pulmonary tuberculosis (67.6%, 10.3%, 22.1% vs. 80.1%, 6.8%, 13%, p < 0.001). The distribution of participants according to whether they were tested for HIV and/or whether such test was positive varied significantly across outcome of care for tuberculosis (Table 1).

### Determinants of the outcomes of care

In sex and age adjusted multinomial logistic regression analysis, and using the combined outcome of cure/treatment completed (treatment success) as the reference, not testing for HIV was positively and significantly associated with treatment default/failure, deaths and transferred-out (Table 2). The range of effect was similar from these 3 broad outcomes. Among those tested for HIV, a positive status was associated with an odds ratio (95% confidence interval) of 6.40 (3.57-11.46) for mortality, but had no effect on other outcomes (Table 2). Similar range of effect was observed when participants with unknown status for HIV were compared with those with negative status [6.49 (3.26-12.93)]. In sex and age adjusted analysis, age was associated with mortality, urban residence with transfer-out, and SPTB- associated with death and treatment default/failure (Table 2).

**Table 2:** Sex and age adjusted predictors of the tuberculosis treatment outcome, odds ratio and 95% confidence interval

	**Cured/completed**	**Default/failure**	**Deaths**	**Tranferred**	**LR test (p-value)**
**Age, per year**	1 (reference)	1.01 (1.00–1.02)	1.05 (1.03-1.06)	1.02 (1.00–1.03)	<0.001
**Sex, men vs. women**	1 (reference)	1.07 (0.83–1.37)	0.91 (0.58-1.43)	0.83 (0.56–1.26)	0.717
**Residence, urban vs. rural**	1 (reference)	1.16 (0.81–1.66)	1.03 (0.55-1.94)	3.56 (2.26–5.62)	<0.001
**Clinical forms**					0.032
**PTB+**	1 (reference)	1 (reference)	1 (reference)	1 (reference)	
**EPTB**	1 (reference)	1.11 (0.79–1.56)	1.00 (0.54–1.86)	0.74 (0.45–1.24)	
**PTB-**	1 (reference)	1.83 (1.13–2.97)	2.42 (1.12–5.25)	1.09 (0.51–2.36)	
**Type of patient, new vs retreatment**	1 (reference)	0.76 (0.51–1.14)	1.59 (0.63–4.06)	1.85 (0.74–4.67)	0.13
**HIV serology**					<0.001
**Negative**	1 (reference)	1 (reference)	1 (reference)	1 (reference)	
**Positive**	1 (reference)	1.19 (0.89–1.59)	6.37 (3.55–11.41)	1.14 (0.70–1.86)	
**Not done**	1 (reference)	2.51 (1.78–3.53)	6.49 (3.26–12.93)	2.87 (1.69–4.86)	
**HIV test, not done vs. done**	1 (reference)	2.37 (1.70–3.28)	2.48 (1.43–4.31)	2.79 (1.69–4.60)	<0.001
HIV serology*, Positive vs Negative	1 (reference)	1.19 (0.89–1.60)	6.40 (3.57–11.46)	1.13 (0.69–1.85)	<0.001

PTB+, smear positive pulmonary tuberculosis ; EPTB, extra-pulmonary tuberculosis ; PTB-, smear negative pulmonary tuberculosis; LR, likelihood ratio ; *only in the subgroup of participants with test done.

In multivariable analysis, with further adjustment for residence and clinical form of tuberculosis, associations of HIV testing and HIV status with the outcome of treatment were largely similar to those observed in age and sex adjusted analysis, with only marginal attenuation of the effect sizes (Table 3). However, the global effect of clinical form of tuberculosis on the outcome of antituberculosis treatment was no longer significant (p-value = 0.140 for the likelihood ratio test).

**Table 3:** Multivariable adjusted predictors of the tuberculosis treatment outcome, odds ratio and 95% confidence interval

	**Cured/completed**	**Default/failure**	**Deaths**	**Tranferred**	**LR test (p-value)**
**Age, per year**	1 (reference)	1.00 (0.99–1.02)	1.04 (1.03-1.06)	1.01 (0.99-1.02)	<0.001
**Sex, men vs. women**	1 (reference)	0.98 (0.76–1.27)	0.89 (0.56-1.41)	0.69 (0.45-1.05)	0.372
**Residence, urban vs. rural**	1 (reference)	1.21 (0.84–1.74)	1.10 (0.58-2.01)	3.81 (2.39-6.06)	<0.001
**Clinical forms**					0.140
**PTB+**	1 (reference)	1 (reference)	1 (reference)	1 (reference)	
**EPTB**	1 (reference)	1.21 (0.85–1.73)	1.08 (0.58–2.02)	0.82 (0.48–1.40)	
**PTB-**	1 (reference)	1.86 (1.13–3.01)	2.08 (0.94–4.60)	1.18 (0.53–2.60)	
**Type of patient, new vs retreatment**	1 (reference)	0.73 (0.48–1.11)	1.43 (0.56–3.68)	1.64 (0.64–4.20)	0.20
**HIV serology**					<0.001
**Negative**	1 (reference)	1 (reference)	1 (reference)	1 (reference)	
**Positive**	1 (reference)	1.19 (0.89–1.59)	6.43 (3.57–11.57)	1.13 (0.95–1.85)	
**Not done**	1 (reference)	2.44 (1.73–3.45)	6.09 (3.02–12.28)	2.76 (1.62–4.72)	
**HIV test, not done vs. done**	1 (reference)	2.30 (1.65–3.21)	2.26 (1.29–3.97)	2.69 (1.62–4.46)	<0.001
HIV serology*, Positive vs Negative	1 (reference)	1.19 (0.88–1.59)	6.35 (3.53–11.45)	1.14 (0.69–1.86)	<0.001

PTB+, smear positive pulmonary tuberculosis ; EPTB, extra-pulmonary tuberculosis ; PTB-, smear negative pulmonary tuberculosis; LR, likelihood ratio; *only in the subgroup of participants with the test done.

### Effects of HIV severity on the outcomes of tuberculosis treatment

**Figure 1:**
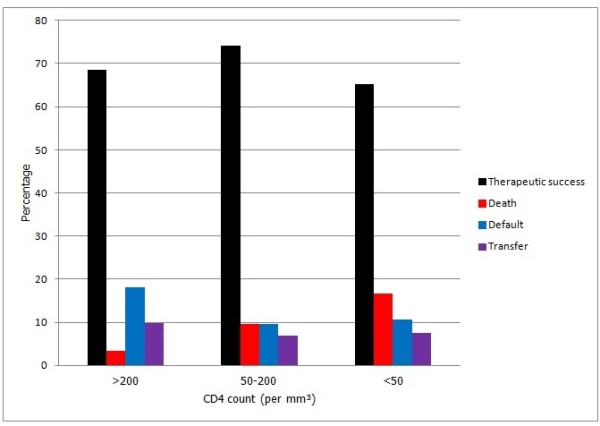
**CD4 count and outcome of tuberculosis treatment among HIV positive patients.** Therapeutic success, Death, Defaulted, Transferred.

A subgroup of HIV positive patient (278) also had CD4 count data available. Figure 1 show the distribution of outcomes of tuberculosis care according to the stage of immune-depression in this subgroup. Mortality rate increased with decreasing CD4 count (CD4 count < 200/mm^3^) while treatment success was better in those with higher CD4 count (p = 0.03, Figure 1).

## Discussion

Our study from this historical diagnosis and treatment centre for tuberculosis in Cameroon has revealed the following major findings: 1) In spite of successive effort and several incentive, about one in eight patients still fails to get screen for HIV during active tuberculosis. 2) Among those tested, about a third is positive for HIV, with women being disproportionately affected. 3) Not testing for HIV or been tested positive are associated with similar excess mortality, and furthermore, not testing is associated with all poor outcomes of tuberculosis treatment.

The co-occurrence of HIV infection and tuberculosis is a well-known fact in Africa. Indeed, the re-emergence of tuberculosis in SSA over the last two decades largely mirrored the explosion of HIV epidemic in this region [4,15]. Most recent studies from Africa suggest that rates of TB/HIV co-infection range from 16% to about 80% [6,7,16-20]. In Asia where tuberculosis is also common, co-occurrence with HIV is found in less than 38% of patients [1,21,22]. In Cameroon, the prevalence of TB/HIV co-infection was 16.6% in 1997 [9], and further increased to 29.3% in 2007 [5] among patients with smear positive pulmonary tuberculosis, at the Yaounde Jamot’s Hospital. This shift mirrors the rising trend of HIV infection at the community level in Yaounde, with prevalence rates increasing from 3% in 1994 [23] to 5.1% in 2007 [24].

The high prevalence of HIV in patients with extra-pulmonary tuberculosis has been described elsewhere [6,25,26]. Our findings therefore are largely in agreement with published literature. Similarly, patients with SPTB- were more likely to be HIV positive than those with SPTB+. Unlike patients with tuberculosis and without HIV, those with HIV are likely to have lung lesions without cavitations [4,27], and accordingly negative smear [28].

We found that not testing for HIV or been tested positive were associated with similar range of risks for all-cause mortality. This in some ways reflects the similarity of profile between patients not tested and those tested positive, and may further suggest that a consistent number of those not tested would have been HIV positive individuals, and perhaps with advanced stage of the disease. Furthermore, those patients who were not tested for HIV were likely those who will default or who will be transferred-out which is also full of significance. One explanation would be that failure to get tested for HIV in this setting is a simple and reliable indicator of future poor adherence to antituberculosis treatment. Another explanation could be that, non-consent for HIV test affects the attitude of the healthcare team toward the patient, with renewed invitations to the patient to accept the test. Under such a pressure, patients would either abandon the treatment or request for their discharge or transfer to another centre.

This study has some limitations. Among the subgroup of patients tested positive for HIV, not everyone had CD4 count, and therefore we were unable to fully account for the possible effect of advanced stage of HIV infection on the outcome of care for tuberculosis. We’ve got no suggestion that missing CD4 count would occur in a differential way to affect the validity of our findings. That no systematic effort was in place to trace patients who dropped out during the year of the study has probably introduced some biases in our ranking of patients according to the outcome of care. For instance, some patients who died in-between antituberculosis treatment drug collection visits would have been inappropriately classified as drop-out. However, the effects of misclassification if any would need to be very important to invalidate our findings.

Our study also has major strengths. These include our large sample size, including the totality of adults patients followed at the CDT during the year of the study. The choice of analytic methods has also allowed us to examine the effects of HIV testing and HIV status on the broad range of outcome of tuberculosis treatment, which is what many previous investigators have not done.

## Conclusions

HIV infection is frequent among patients with active tuberculosis in this setting and disproportionately affects women. Both not testing for HIV and been tested positive confer a high risk of mortality in our patients with tuberculosis. In addition, not testing for tuberculosis is associated with poor outcomes of tuberculosis. Efforts are needed to improve the acceptance of HIV testing among patients with tuberculosis and reduce mortality risk among those tested positive in our context.

## Abbreviations

AFB: Acid-fast bacilli; CDT: Centre for diagnosis and treatment of tuberculosis; E: Éthambutol; EPTB: Extra-pulmonary tuberculosis; H: Isoniazid; HIV: Human immunodeficiency virus; NPAT: National programme against tuberculosis; PTB-: Smear-negative pulmonary tuberculosis; PTB+: Smear-positive pulmonary tuberculosis; R: Rifampicin; S: Streptomycin; SSA: Sub-Saharan Africa; TB: Tuberculosis; YJH: Yaounde Jamot’s Hospital; Z: Pyrazinamide.

## Competing interests

The authors declare that they have no competing interests.

## Authors’ contributions

EWPY conceived the study, supervised data collection, co-analysed the data and drafted of the manuscript; CK supervised the data collection and critically revised the manuscript; APK contributed to study designed, data analysis, drafting and critical revision of the manuscript. All authors read and approved the final manuscript.

## Pre-publication history

The pre-publication history for this paper can be accessed here:

http://www.biomedcentral.com/1471-2334/12/190/prepub
